# Design and Synthesis of New Thiophene/Thieno[2,3-*d*]pyrimidines along with Their Cytotoxic Biological Evaluation as Tyrosine Kinase Inhibitors in Addition to Their Apoptotic and Autophagic Induction

**DOI:** 10.3390/molecules27010123

**Published:** 2021-12-26

**Authors:** Elshaymaa I. Elmongy, Nashwah G. M. Attallah, Najla Altwaijry, Manal Mubarak AlKahtani, Hanan Ali Henidi

**Affiliations:** 1 Department of Pharmaceutical Sciences, College of Pharmacy, Princess Nourah bint Abdulrahman University, Riyadh P.O. Box 84428, Saudi Arabia; ngmohamed@pnu.edu.sa (N.G.M.A.); naaltwaijry@pnu.edu.sa (N.A.); 2 Department of Pharmaceutical Chemistry, Faculty of Pharmacy, Helwan University, Ain Helwan, Cairo P.O. Box 11795, Egypt; 3 Egyptian Drug Authority (EDA) (Previously NODCAR), Giza 8655, Egypt; 4 Research Department, Health Sciences Research Center, Princess Nourah bint Abdulrahman University, Riyadh P.O. Box 84428, Saudi Arabia; mamalkahtani@pnu.edu.sa (M.M.A.); hahenidi@pnu.edu.sa (H.A.H.)

**Keywords:** thieno[2,3-*d*]pyrimidines, protein kinases, cytotoxicity, flow cytometry

## Abstract

This work describes the synthesis and anticancer activity against kinase enzymes of newly designed thiophene and thieno[2,3-*d*]pyrimidine derivatives, along with their potential to activate autophagic and apoptotic cell death in cancer cells. The designed compounds were scanned for their affinity for kinases. The results were promising with affinity ranges from 46.7% to 13.3%. Molecular docking studies were performed, and the compounds were then screened for their antiproliferative effects. Interestingly, compounds **8** and **5** resulted in higher cytotoxic effects than the reference standard against MCF-7 and HepG-2. The compounds were evaluated for their induction of apoptosis and/or necrosis on HT-29 and HepG-2. Three compounds induced significant early apoptosis compared to untreated control HT-29 cells, and four derivatives were more significant compared to untreated HepG-2 cells. We further investigated the effect of four compounds on the autophagy process within HT-29, HepG-2, and MCF-7 cells with flow cytometry. Similar to the apoptosis results, compound **5** showed the highest autophagic induction among all compounds. The potential inhibitory activity of the synthesized compounds on kinases was assessed. Screened compounds showed inhibition activity ranging from 41.4% to 83.5%. Compounds recorded significant inhibition were further investigated for their specific FLT3 kinase inhibitory activity. Noticeably, Compound **5** exhibited the highest inhibitory activity against FLT3.

## 1. Introduction

We are living in an exciting era when research is being turned into real-world improvements in diagnosis, prevention, and therapy. Developing novel compounds that can be used to cure cancer is one of the core interests of scientific research. The anticancer impact of produced heterocyclic compounds is well recognized as one of the most important qualities that can be used in therapeutic science [1]. Heterocyclic ring structures containing the thienopyrimidine moiety are of interest due to their varied pharmacological and biological properties [2,3,4]. They are bio-isosteres to purine with a thiophene ring intertwined with pyrimidine. This platform has become an exciting basic component in the development of pharmaceutical agents due to its broad variety of applications [5,6]. Thienopyrimidines have been innovated as compounds with many interesting activities including as antioxidants [7], anticancer agents [8,9,10], and tyrosine kinase inhibitors [10,11,12].

Most of the synthesized anticancer compounds have an heterocyclic core derivatized from pyrimidine, and many of them are thienopyrimidines. Interestingly, they have recently been investigated as scaffolds for protein kinase (PKs) inhibitors [10,11,12,13,14,15,16].

Protein kinases are protein phosphorylating enzymes responsible for regulating the progression, division, and proliferation sequence of cell cycle events [17]. They cause phosphorylation of amino acids leading to conformational changes which form active proteins. Accordingly, they are known to regulate protein-biological activity [18,19]. Protein kinases have a role in carcinogenesis when they are expressed in mutant, uncontrolled forms, or when they are produced in excessively high quantities, and they can transform normal cells into cancerous phenotypes [20]. Protein tyrosine kinases (PTKs) not only aids in tumor angiogenesis and metastasis, but it also helps to regulate cell division [21].

An interesting member of protein kinases is Fms-like tyrosine kinase 3 (FLT3). FLT3 is a class III membrane-bound tyrosine kinase (RTK) receptor expressed by hematopoietic progenitors [22,23]. FLT3 supports hematopoietic stem cells survival and its proliferation as well when activated by its ligand (FLT3L) and is important to maintain normal stem cell development [24].

Emerging from the literature [25], thienopyrimidine-based analogues were developed by modifying the IKKβ inhibitor “SPC-839” that was pharmacologically tested revealing a promising inhibitory profile against FLT3 [13]. Yet recently, a series of thieno[2,3-*d*]pyrimidines were synthesized and tested for their FLT3 kinase inhibition activity in addition to its proliferation activity on four human cell lines, resulting in significant data [14].

**Figure 1:**
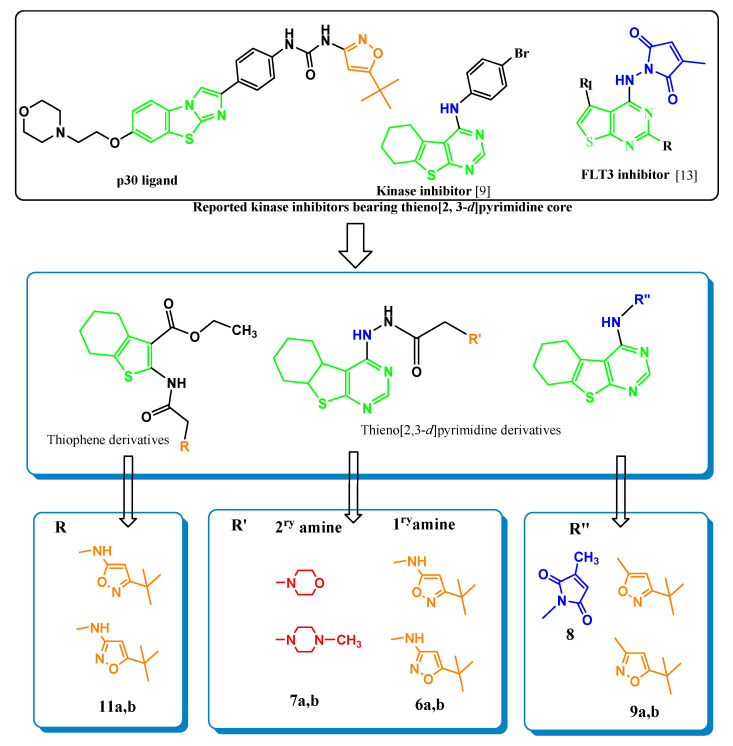
Primary designed strategy for new thieno[2, 3-*d*]pyrimidine series.

Motivated by the above-mentioned facts, herein we designed and synthesized a series of thiophene/thienopyrimidines and then evaluated their antiproliferative and kinase inhibitory activity (Figure 1). Structural modifications were carried out on thienopyrimidines of a promising kinase inhibitory profile. Introduced thienopyrimidine as a core and different functional groups were incorporated at position 4. This derivatization is also supported by recent reports of kinase inhibitors bearing thieno[2,3-*d*]pyrimidine fragments [13,14]. Moreover, compounds with thiophene core were synthesized, and substituents were added at position 2. Groups introduced to the thiophene and thienopyrimidne ring had been previously incorporated in reported active kinase inhibitors, they are either the methyl pyrrole-2,5-dione moiety [13,14] or the primary amine “3-*tert*-butyl isoxazal-5-yl amine which is presented in the enzyme ligand P30”. Then secondary amines namely morpholine and *N*-methyl piperazine were used instead of the primary amines, aiming to determine their effect on biological activity, Figure 1. The synthesized compounds were then evaluated for their cytotoxic activity *in*-*vitro* against three cell lines in addition to their kinase inhibitory activity and FLT3 kinase inhibitory activity as well. Compounds that showed the best results were subjected for their apoptosis /necrosis assessment using flow cytometric analysis.

## 2. Results and Discussion

### 2.1. In Silico-Target Prediction

**Figure 2:**
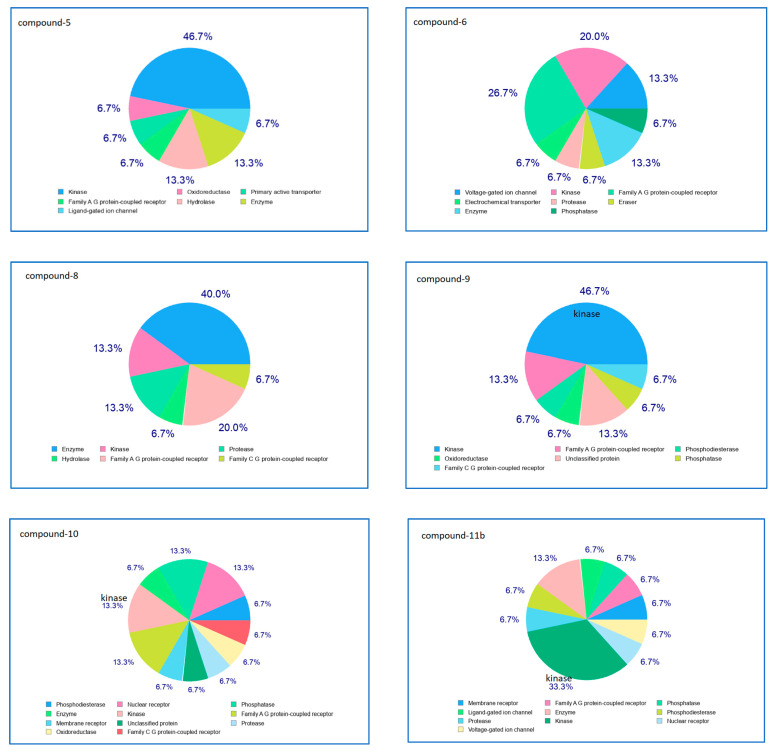
Percentage affinity of the designed compounds for kinases rather than other targets.

Compounds were scanned using Swiss Target Prediction software which showed promising affinity for kinases, in particular FLT3 kinase. The highest percentage 46.7% was recorded by compounds **5** and **9** while compound **11** expressed 33.3% kinase affinity. Moreover, compounds **6** and **10** showed kinase affinity equivalent to 20 and 13.3%, respectively, Figure 2.

### 2.2. Molecular Docking

**Figure 3:**
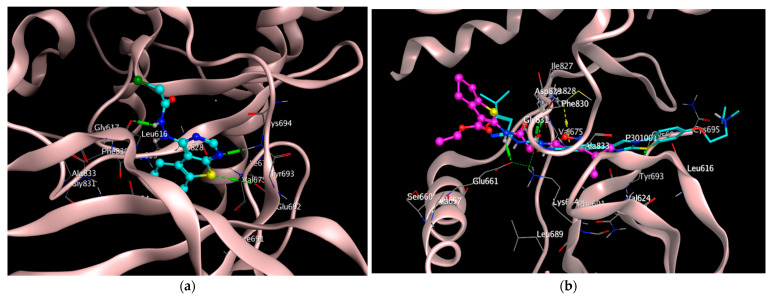
Binding mode of the least RMSD compound **5** with thienopyrimidine nucleus and the lowest binding energy compound **11b** with thiophene core at target active site. (**a**) Compound **5** (cyan) totally inside target pocket, aminoacids involved in interactions are Leu 616, Cys 694, Glu 692. (**b**) Compound **11b** (pink) overlap with the protein ligand **P30** (cyan). Interaction with active site involved H-bonding interaction with Glu 661, Asp 829 in addition to hydrophobic interaction with Cys 828.

Molecular docking studies were performed to investigate the affinity of the prepared compounds to FLT3 kinase enzyme which was expressed in an interesting value in the above-mentioned target-affinity prediction. The crystal structure of FLT3 kinase bound to its ligand P30 was downloaded from Protein Data Bank (PDB:4XUF). Docking simulation of the synthesized compounds at FLT3 active site was carried out in comparison with the co-crystalized ligand P30 downloaded from protein data bank (PDB: 4XUF). For validation of results, redocking of the crystalized ligand was carried out recording RMSD deviation of 1.247 **A**. Results of docking, regarding binding energy and root mean square deviation RMSD in addition to the ligand interactions are tabulated below. Amino acids involved in the interaction between the co-crystalized ligand and the active site were Glu 661, Asp 829, Leu 616, and Cys 694. These interactions were either hydrogen bonding or hydrophobic interactions Figure 3a,b. These amino acids interactions were also in common between the synthesized compounds and the active ligand site mainly at Leu 616 and Cys 694 as illustrated in Table 1. Although compound **11** showed the least binding energy to the FLT3 pocket (−9.01 kcal/mol) and it has a thiophene core structure, but compounds with thiophene ring were in general less in biological activity than those with thienopyrimidine nucleus. Those with thienopyrimidine core having substitutions added at position 4 especially **5**, **8**, and **9** recording binding scores −8.068, −7.529, and −8.360, respectively, are the most significant biologically either in the preliminary antiproliferative screening or kinase inhibition. Interestingly, the chloro-acetohydrazide derivative of thienopyrimidine **5** expressed the lowest RMSD value, with 0.79 **A** showing the most potent cytotoxic effects against all cell lines and exhibiting the highest inhibitory activity against FLT3 with IC_50_ 32.435 ± 5.5 μM.

### 2.3. Chemistry

#### 2.3.1. Synthesis

**Scheme 1:**
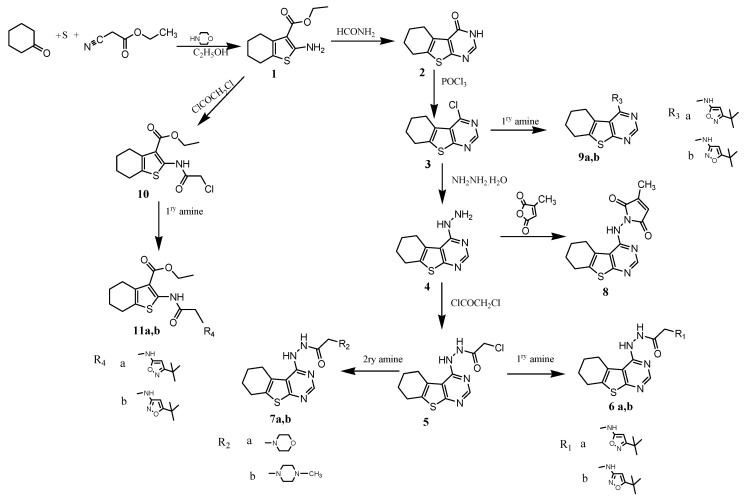
Synthesis pathways for compounds **1**–**11**.

The synthetic strategy to synthesize the target thienopyrimidines **1–11** is depicted in Scheme 1. The aminothiophene carboxylate ester **1** was prepared through reported procedures of Gewald reaction using the proper cycloketone, ethyl cyanoacetate, sulfur, and a secondary amine [26,27].

Refluxing **1** with excess formamide as reported [26,28] gave the cycloheptathieno[2,3-*d*]pyrimidin-4(3*H*)-one derivative **2** which yielded the corresponding para chlorinated derivative **3** upon treatment with phosphorus oxychloride as described in the literature [29]. Hydrazine hydrate in ethanol was then refluxed with **3** to produce the 4-hydrazino **4** [29] which upon treatment with chloroacetyl chloride under gentle heating for 4 h afforded the chloroacetamido derivative **5**. Reaction under reflux of compound **5** with 1^ry^ amines, namely 5-*tert*-butylisoxazol-3-amine and 3-*tert*-butylisoxazol-5-amine in methylene chloride with few drops of triethylamine, resulted in compounds **6a** and **6b**, respectively. Moreover, compounds **7a** and **7b** were prepared upon reaction of the chloro acetamido derivative **5** with the appropriate 2^ry^ amine under reflux for 5 h in dichloromethane (DCM).

The pyrrolo-dione derivative **8** was obtained upon heating compound **5** with 3-methylfuran-2,5-dione. Furthermore, compounds **9a** and **9b** were prepared upon refluxing the *p*-chloropyrimidine derivative **3** with the appropriate 1^ry^ amine namely 5-*tert*-butylisoxazol-3-amine and 3-*tert*-butylisoxazol-5-amine, respectively, in chloroform for 9 h. Reaction of the aminothiophene carboxylate **1** with chloroacetyl chloride yielded the acetamido derivative **10**, which upon refluxing with the appropriate 1^ry^ amine (namely 5-*tert*-butylisoxazol-3-amine and 3-*tert*-butylisoxazol-5-amine, respectively) in chloroform overnight afforded **11a** and **11b**, respectively.

#### 2.3.2. Structural Confirmation

Structural characterization of the synthesized compounds was confirmed by ^1^H-NMR and ^13^C-NMR in addition to elemental analysis. ^1^H-NMR for **5** revealed two exchangeable protons at δ 4.00 and 12.00 ppm and ^13^C-NMR reported (C = O) group at δ 165.3 ppm. The isoxazole derivatives **6** were confirmed from the newly added singlet standing for NH that was D_2_O exchangeable in addition to the appearance of C = C and C = N at δ 152.6, 155.1 ppm, respectively, in ^13^C-NMR. The acetohydrazides **7** showed two exchangeable NH protons in addition to increased number of carbons illustrated in ^13^C-NMR at a range between δ 5 to 6 ppm for the newly appeared morpholine for **7a** and piperazine for **7b**. Moreover, the pyrrolodione derivative **8** showed the NH group at δ 4.21 ppm which disappeared upon deuteration and appearance of two C = O signals at δ 164.00 and 165.29 ppm in its ^13^C-NMR. ^1^H-NMR for the pyrimidine 4-aminoisoxazole derivatives **9** confirmed an exchangeable NH group at δ 4.00 ppm. In addition, *tert*-butylisoxazol-thiophene-3-carboxylate **11** displayed two D_2_O exchangeable NH signals at δ 3.80 and 8.12 ppm and a peak at δ 4.30 ppm standing for acetamide protons, (Scheme 1).

### 2.4. Biological Activity

#### 2.4.1. Antiproliferative/Cytotoxicity Assessment

**Figure 4:**
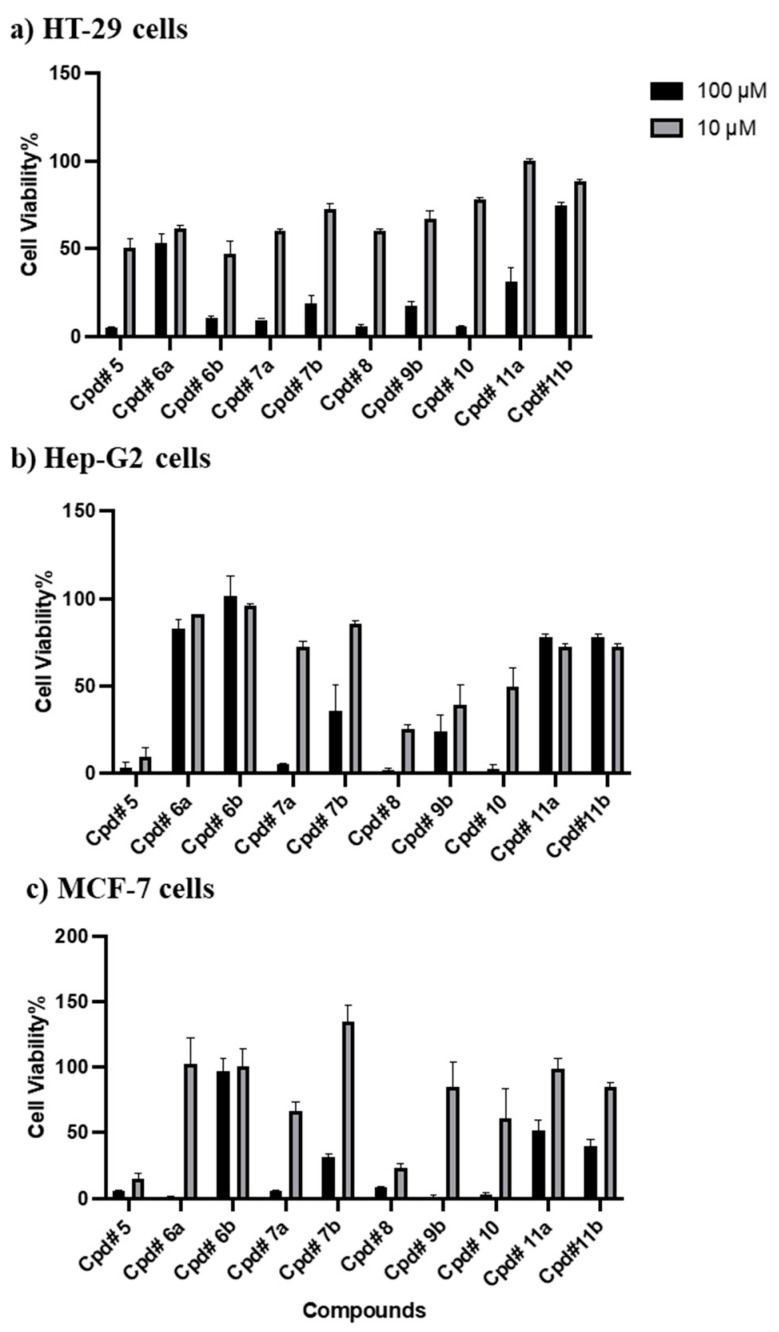
Effect of synthesized compounds on the viability of HT-29 (**a**), HepG-2 (**b**), and MCF-7 (**c**). Cells were treated with 10 µM and 100 µM for 72 h and viability was determined using MTT assay. Data are expressed as mean ± SD; *n* = 3.

The synthesized compounds were subjected to in vitro proliferative assay against HT-29 (Colorectal Adenocarcinoma), HepG-2 (Hepatocellular carcinoma), and MCF-7 (breast adenocarcinoma). All compounds were exposed to 10 and 100 μM concentrations for 72 h. The mean inhibition percentages of all compounds over the tested cell lines are shown in Figure 4. All synthesized compounds showed moderate to significant antiproliferative activity against cancer cell lines in 100 µM concentration. On the other hand, the mean inhibition after exposure to concentration 10 µM was strong in compounds **5** and **8**, moderate in compounds **7a**, **9b**, and **10**, and low in compounds **6a**, **6b**, **7b**, **11a**, and **11b** Figure 4. By correlating the structural variations and the resulted antiproliferative activities, we can conclude that compounds with thienopyrimidine core having substitutions added at position 4, are the most significant biologically as in compounds **5**, **8**, and **9**. The chloro-acetohydrazide derivative of thienopyrimidine **5** showed the most potent cytotoxic effects against all cell lines followed by the thienopyrimidine pyrrole-2,5-dione derivative **8** as they exhibited more than 50% drop in cell viability at 10 μM concentration, compared to the control. Here we might reveal the activity of compound **8** to the incorporation of methyl pyrrolodione to the thienopyrimidine nucleus. In addition, introduction of the *tert*-butyl isoxalyl amine in compound **9** led to moderate antiproliferative activity among tested compounds. However, introducing a spacer between thienopyrimidine C-4 and the amino substituent led to drop in their antiproliferative activity as shown in compounds **6a**, and **6b** which had the primary amine “3-*tert*-butyl isoxazal-5-yl amine” and “5-*tert*-butyl isoxazal-3-yl amine”, respectively. Incorporating secondary amine namely, morpholine and *N*-methyl piperazine instead of the primary amine compounds **7a** and **7b** led to an increase in activity specially for compound **7a** which exhibited about 40% drop in cell viability at 10 μM in HT-29 and MCF-7 cell lines. Compounds with thiophene ring were less in biological activity than those with thienopyrimidine nucleus although compounds **11a** and **11b** showed the least binding energy to the FLT3 pocket.

##### Detailed Dose-Response Relationship against Cancer Cells

After evaluating the preliminary antiproliferative effects of the synthesized compounds, compounds showing good potency were further assessed to calculate IC_50_ values against tested cell lines (HT-29, HepG-2, and MCF-7). The anti-proliferative activities of compounds **5**, **7a**, **8**, **9b**, and **10** were compared with Doxorubicin as a positive control. As shown in Table 2, the selected compounds showed considerable potency over almost all tested cell lines. However, the results were so much interesting in compounds **5** and **8**. The two compounds **5** and **8** showed high efficacies against MCF-7 with IC_50_’s 7.301 ± 4.5 and 4.132 ± 0.5 μM, respectively, also against HepG-2 with IC_50_’s 5.3 ± 1.6 and 3.3 ± 0.90 μM, respectively. Interestingly, HT-29 and MCF-7 cells survived at much higher concentrations of tested compounds compared to HepG-2 which may indicate the specificity of these compounds Table 2. However, it was reported that cancer cells are associated with overexpression/deregulation of kinases [30]. Thus, the ability to target kinase activity represents an attractive therapeutic strategy for cancer treatment [30]. However, similar thieno[2,3-*d*]pyrimidine derivatives were reported remarkable selectivity toward cancer cells as compared to normal cells [31,32].

#### 2.4.2. Apoptosis/Necrosis Assessment

**Figure 5:**
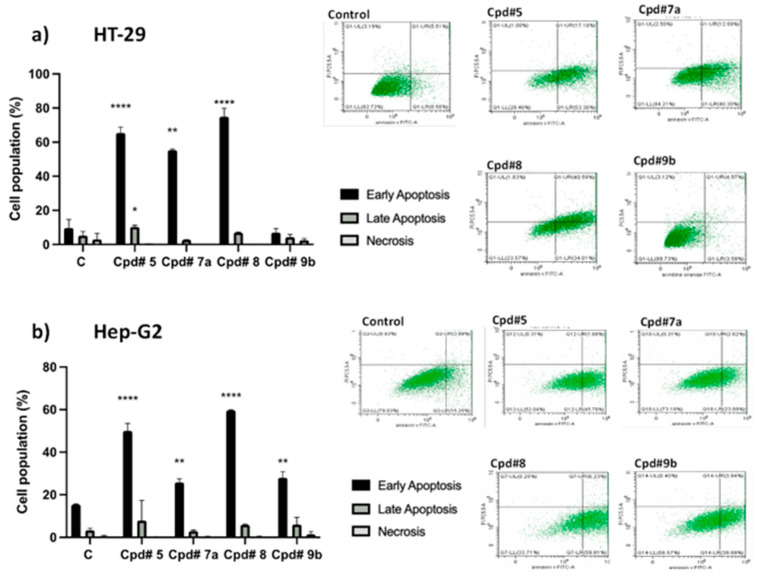
Apoptosis/necrosis assessment in HT-29 (**a**) and HepG-2 (**b**) cells after exposure to the synthesized compounds. Cells were exposed to predetermined IC_50_ of compounds **5**, **7a**, **8**, and **9b** for 24 h and were stained with annexin V-FITC/PI. Cell populations were plotted as percentage of total events. Data is presented as mean ± SD; *n* = 3. * Significantly different from the control group at *p* < 0.05. ** Significantly different from the control group at *p* < 0.005 **** Significantly different from the control group at *p* < 0.0001.

Herein, we examined the effect of selected compounds for inducing further apoptosis and necrosis. HT-29 and HepG-2 cells were exposed to the pre-calculated IC_50_ values of **5**, **7a**, **8**, and **9b** for 24 h rather than 72 h to detect early apoptotic populations. In HT-29 cells, compounds **5**, **7a**, and **8** induced significant early apoptosis 65.3 ± 3.4%, 55.3 ± 0.70%, and 74.9 ± 4.9%, respectively, compared to untreated cells 9.6 ± 4.9%. Similarly, the exposure of HepG-2 cells to **5**, **7a**, **8**, and **9b** resulted in a significant increase in apoptosis 49.9 ± 3.5%, 25.8 ± 1.6%, 59.6 ± 0.19%, and 27.9 ± 2.9%, respectively, compared to untreated cells 15.4 ± 0.25%. Among all compounds examined, compound **5** induced a significant increase in the late apoptotic cell population in HepG-2 and HT-29 cells by 2.3 and 1.9-fold, respectively. By contrast, none of the compounds induced any significant increase on the necrotic cell population. Hence, the cytotoxic effects of these compounds against HepG-2 and HT-29 cells are mostly attributed to apoptosis rather than necrosis Figure 5.

#### 2.4.3. Autophagy Assessment

**Figure 6:**
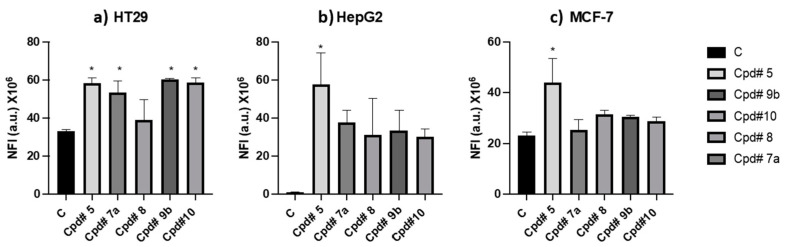
Autophagic cell death assessment in HT-29 (**a**) HepG-2 (**b**) and MCF-7 (**c**). Cells after exposure to compounds **5**, **7a**, **8**, **9b**, and **10** for 24 h. Cells were stained with acridine orange staining. Net fluorescent intensity (NFI) was counted and compared to the basal fluorescence of the control group. Data are presented as mean ± SD; *n* = 3. * significantly different from the untreated cells.

During the autophagic process, the formation of acidic vesicular organelles (AVOs) is one of the characteristics of autophagic cells in response to different inducer agents [33,34]. Herein, we further investigated the induction of cellular acidification within HT-29, HepG-2, and MCF-7 cell lines by using acridine orange staining coupled with flow cytometry. The percentage of net fluorescent intensities (NFI) for AVOs formation was calculated after exposure to the pre-determined IC_50_ of selected compounds **5**, **7a**, **8**, **9b**, and **10**. In HT-29 cells, compounds **5**, **7a**, **9b**, and **10** significantly induced the formation of AVOs at tested dose by 58.11 ± 2.8%, 53 ± 6.07%, 60.3 ± 0.4%, and 58.7 ± 2.4%, respectively, Figure 6. Similar to what was observed with apoptosis, compound **5** showed the highest autophagic induction among all compounds and significantly increased autophagic signal in HepG-2 and MCF-7 cells by 57.5 ± 16.8% and 44.07 ± 9.3%, respectively. These findings indicate that compound **5** has the potential to activate autophagic and apoptotic cell death in cancer cells. In fact, it has been suggested that tyrosine kinase inhibitors (TKIs) are able to induce autophagy independent of their original target molecules [35,36].

### 2.5. Kinase Inhibition Activity

**Figure 7:**
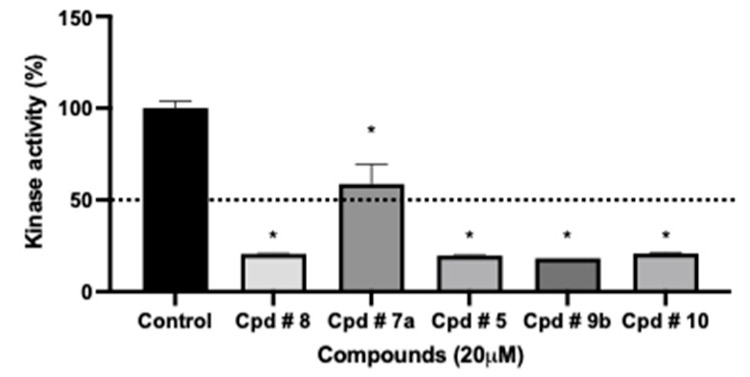
The effect of test compounds at 20 μM concentration against the activity of total intracellular kinase enzymes. Data are expressed as mean ± SD; *n* = 3. * Significantly different from control group (*p* value < 0.0001).

The initial screening was performed to determine the preliminary kinase inhibition activity of the synthesized compounds. screened compounds showed inhibition activity at the testing dose (20 μM) ranging from 41.4% to 81.8%. Like what was predicted in kinase affinity, compounds **5**, **8**, **9b**, and **10** recorded significant activity with % kinase enzyme inhibition ranging from 79.4% to 81.8%, Figure 7. Compound **7a** showed mild kinase inhibition of 41.4%. Observed agreement between kinase inhibition and antiproliferative activity can be further assessed to identify the potential inhibitors of oncogenic receptors including FLT3 receptor. However, similar to our findings, previous studies reported that compounds bearing the thieno[2,3-*d*]pyrimidines scaffold exhibited inhibitory activity against kinases [31,37].

#### FLT3 Screening

Compounds with the high potency at tested cancer cell lines and possess kinase inhibitory profiles **5**, **8**, **9b**, and **10** were screened for their FLT3-kinase inhibitory activity. Compounds **5** exhibited moderate selectivity for FLT3 with an IC_50_ 32.435 ± 5.5 μM. As shown in Table 3, compound **8** exhibited inhibitory activity comparable to compounds **9b** and **10** with IC_50_’s 40.55 ± 6.3, 39.61 ± 4.6 and 40.04 ± 5.5 μM, respectively. Referring to the affinity of the compounds to FLT3 kinase and ligand interactions data summarized in Table 1, compound **5** expressed the lowest RMSD with 0.79 **A** among the other compounds, also amino acids involved in the main interactions between the protein ligand (P30) and the active site were also in common between the synthesized compounds and the active ligand site. However, the moderate activity of compounds **5**, **8**, **9b**, and **10** over FLT3 kinase may not justify their high efficacies and wide spectrum antiproliferative activities in cancer cells. This finding suggests the existence of other underlying mechanisms that trigger the anticancer activity of these new compounds in addition to their mild FLT3 kinase inhibition.

### 2.6. Bioavailability and Drug Likeness Investigation

Best biological scoring compounds **5**, **8**, **9**, and **10** in addition to compound **11** the best scoring binding energy towards the target, were subjected for bioinformatics assessment [11]. The investigated compounds had good well-permeability and absorption as tabulated below, Table 4.

Compounds had between four and six hydrogen-bond donor (HBD) and one or two hydrogen-bond acceptors (HBA). In addition, all of the tested compounds had strong toleration by cell membranes as they recorded log *P* range < 5 except compound **9** which recorded (5.07) which is slightly above the limit [38]. Regarding drug-likeness scores, compounds are considered with drug-like properties when they express positive value. All of the tested compounds showed positive values, best of which is compound **8** that recorded 0.98, Table 4. Interestingly, all of the investigated molecules had log *P* ≤ 5, molecular weight ≤ 500, number of hydrogen bond acceptors ≤ 10, and number of hydrogen bond donors ≤ 5 and blood brain barrier (BBB) score ranging from 3.2 to 3.54 which is considered a very promising value range. Hence, in reference to Lipinski’s rule of five [11] and according to the above-mentioned data, all of the investigated compounds are considered good candidates as “drug-like” molecules with promising bioavailability.

## 3. Experimental

### 3.1. Molecular Modeling Study

The crystal structure of FLT3 kinase bound to its ligand was downloaded from Protein Data Bank (PDB:4XUF). Protein optimization: amino acids residues were optimized, water molecules and ions were deleted, and hydrogens were added.

Optimization of ligands chemical structures: all compounds were built using MOE 2014 builder, the bond order adjusted, partial charges were added, as well as hydrogens., energy was minimized, and compounds were then saved as mol2 format.

Docking protocol applied was induced fit to allow the receptor to move during refinement. Ligand atoms were chosen to define the active site, alpha spheres with 5 **A** were used to guide the placement. Pharmacophore annotations were excluded. Ligand was defined as the MDB file and rotatable bonds were allowed. The docking placement methodology was the triangle matcher, the initial scoring methodology was London dG, a forcefield was applied as the post placement refinement, and duplicate poses were removed. The gradient for energy minimization was set to be = 0.05, and the force field was selected as “MMFF94X” to be suitable for organic molecules. All bonded, Van der Waals, electrostatics, and restraints were enabled. Partial charges were calculated. Best scoring was chosen depending on binding energy and RMSD values.

### 3.2. Chemistry

Melting points were determined on Stuart SMP10 apparatus and the values were uncorrected. A Bruker advance 400 MHz NMR spectrometer was used for ^1^H NMR spectral analysis in Cairo University Microanalytical Unit, Faculty of Pharmacy, Egypt. Tetramethylsilane (TMS) was used as internal standard upon recording Chemical shift values in ppm on d scale using. ^13^C NMR spectra were carried out in Cairo University Microanalytical Unit, Faculty of Pharmacy using a Bruker Advance 100 MHz spectrometer, Egypt. C, H, N microanalytical data analysis was performed at the Al-Azhar University Regional Center for Mycology and Biotechnology, Egypt. Biological evaluation was performed at Research Department, Health Sciences Research Center, Princess Nourah bint Abdulrahman University, Saudi Arabia.

The starting materials, namely, **1**, **2**, **3**, **4** and **10** were prepared following the corresponding literature procedures [29].

*2-Chloro-N′-(5,6,7,8,tetrahydrobenzo[4,5]thieno[2,3-d]pyrimidin-4-yl)acetohydrazide*: **5**. Reaction of 1.0 mmol of **4** with 3.0 mmol of chloroacetyl chloride under gentle heating for 4 h in DCM using 4 drops of TEA. The product was filtered, dried, and recrystallized from ethanol. Yield: 80%, m.p. >250 °C; ^1^H-NMR (400 MHz, DMSO-d6) ppm: δ 1.72–2.98 (m, 8H, cyclohexane), 4.00 (s, 1H, NH, D_2_O exchangeable), 4.50 (s,2H, CH_2_), 7.49 (s, 1H, CH pyrimidine), 12.00 (s, NH, D_2_O exchangeable); ^13^C-NMR (100 MHz, DMSO-d6) ppm: δ 24.34, 24.54, 25.00, 47.23, 117.11, 127,60, 137.24, 145.31, 154.45, 163.02, 165.30, 166.24; Anal. for C_12_H_13_ClN_4_OS (296.05), Calcd %: C, 48.56; H, 4.42; N, 18.88. Found %: C, 48.68; H, 4.44; N, 18.39.

*General procedures for compounds***6a***and***6b**. Equimolar amounts of the acetamide derivative **5** and the appropriate 1^ry^ amine namely 5-*tert*-butylisoxazol-3-amine/3-*tert*-butylisoxazol-5-amine, respectively, were stirred under reflux in methylene chloride with few drops of triethylamine for 5 h to afford compounds **6a** and **6b**, respectively.

*2-(5-Tert-butylisoxazol-3-ylamino)-N′-(5,6,7,8-tetrahydrobenzo[4,5]thieno[2,3-d]pyrimidin-4-yl)acetohydrazide*: **6a**. Yield: 70%, m.p. 210–212 °C. ^1^H-NMR (400 MHz, DMSO-d6) ppm: δ 1.50 (m, 9H, t-butyl), 1.60–3.00 (m, 8H, cy-clohexane), 2.13 (s, 1H, isoxazole ring), 4.50 (s, 1H, NH D_2_O exchangeable), 4.95 (s, 2H, CH_2_-NH), 7.00 (s, 1H, NH, D_2_O exchangeable), 8.56 (s, 1H, C-2 pyrimidine), 12.10 (s, 1H, NH D_2_O exchangeable).; ^13^C-NMR (100 MHz, DMSO-d6) ppm: δ 23.3, 24.2, 24.7, 25.3, 33.1, 33.1, 34.4, 35.1, 57.5, 97.3, 117.4, 126.3, 140.1, 147.4, 155.2, 155.6, 167.0, 168.0; Analysis for: C_19_H_24_N_6_O_2_S (400.17): CHN calcd. C, 56.98; H, 6.04; N, 20.98; found: C, 57.03; H, 6.08; N, 21.07.

*2-(3-Tert-butylisoxazol-5-ylamino)-N′-(5,6,7,8-tetrahydrobenzo[4,5]thieno[2,3-d]pyrimidin-4-yl)acetohydrazide*: **6b**. Yield: 70%, m.p. 198–200 °C. ^1^H-NMR (400 MHz, DMSO-d6) ppm: δ 1.50 (m, 9H, t-butyl), 1.62–2.98 (m, 8H, cyclohexane), 2.13 (s, 1H, isoxazole ring), 4.00 (s, 1H, NH D_2_O exchangeable). 4.95 (s, 2H, CH_2_-NH), 6.80 (s, 1H, NH, D_2_O exchangeable), 8.00 (s, 1H, C-2 pyrimidine), 11.32 (s, 1H, NH D_2_O exchangeable); ^13^C-NMR (100 MHz, DMSO-d6) ppm: δ 23.5, 23.8, 24.3, 25.3, 32.3, 32.3, 32.3, 34.1, 57.0, 95.1, 117.4, 126.3, 138.5, 145.4, 152.6, 155.1, 166.4, 168.0; Analysis for: C_19_H_24_N_6_O_2_S (400.17): CHN calcd. C, 56.98; H, 6.04; N, 20.98; found: C, 56.96; H, 6.03; N, 21.02.

*General procedures for compounds***7a***and***7b**. Reaction of the chloro acetamido derivative **5** with the proper 2^ry^ amine morpholine and *N*-methyl piperazine, respectively, under reflux for 5 h in DCM to give compounds **7a** and **7b**, respectively.

*2-Morpholino-N′-(5,6,7,8-tetrahydrobenzo[4,5]thieno[2,3-d]pyrimidin-4-yl)acetohydrazide*: **7a**. Yield: 66%, m.p. 222–224 °C; ^1^H-NMR (400 MHz, DMSO-d6) ppm: δ 1.50–2.55 (m, 8H, cyclohexane), 2.40–3.51 (m, 8H-morpholine ring), 4.00 (s, 2H, CH_2_), 4.50 (s, 1H, NH D_2_O exchangeable) 8.00 (s, 1H, CH-pyrimidine), 12.01 (s, 1H, NH D_2_O exchangeable); ^13^C-NMR (100 MHz, DMSO-d6) ppm: δ 23.5, 23.8, 24.5, 25.3, 55.1, 55.4, 59.1, 66.1, 66.1, 117.7, 126.0, 139.8, 145.4, 154.8, 167.2, 169.2; Analysis for: C_16_H_21_N_5_O_2_S (347): CHN calcd. C, 55.31; H, 6.09; N, 20.16; found: C, 55.33; H, 6.12; N, 20.19.

*2-(4-Methylpiperazin-1-yl)-N′-(5,6,7,8-tetrahydrobenzo[4,5]thieno[2,3-d]pyrimidin-4-yl)acetohydrazide*: **7b**. Yield: 59%, m.p. 214–216 °C; ^1^H-NMR (400 MHz, DMSO-d6) ppm: δ 1.77–2.55 (m, 8H, cyclohexane), 2.24 (s, 3H, CH_3_ piperazine), 3.00–3.50 (m, 8H, piperazine ring), 4.00 (s, 2H, CH_2_), 5.50 (s, 1H, NH D_2_O exchangeable), 8.00 (s, 1H, CH-pyrimidine), 9.00 (s, 1H, NH D_2_O exchangeable); ^13^C-NMR (100 MHz, DMSO-d6) ppm: δ 22.4, 24.3, 24.5, 25.8, 44.3, 55.4, 55.4, 56.3, 56.6, 59.2, 117.7, 127.3, 139.8, 157.8, 163.2, 167.6, 170.0; Analysis for: C_17_H_24_N_6_OS (360): CHN calcd. C, 56.64; H, 6.71; N, 23.31; found: C, 56.59; H, 6.70; N, 23.35.

*3-Methyl-1-(5,6,7,8-tetrahydrobenzo[4,5]thieno[2,3-d]pyrimidin-4-ylamino)-1H-pyrrole-2,5-dione*: **8**. Reaction of 1.0 mmol of **4** with 1.5 mmol of 3-methylfuran-2,5-dione in 15 mL CHCl_3_ for 18 h to give the pyrrolo-dione derivative as yellow powder in Yield: 82%, m.p. <250 °C; ^1^H-NMR (400 MHz, DMSO-d6) ppm: δ 1.72–2.98 (m, 8H, cyclohexane), 2.13 (s, 3H, CH_3_ at C2-pyrrol), 4.21 (s, NH, D_2_O exchangeable), 6.98 (s, 4H, 1H, pyrrole ring), 7.24 (s, 1H, CH pyrimidine), ^13^C-NMR (100 MHz, DMSO-d6) ppm: δ 24.54, 24.54, 25.00, 27.03, 113.33, 122.68, 127,60, 134.55, 137.50, 143.34, 145.71, 154.65, 164.00, 165.29, 168.21; Anal. for C_15_H_14_N_4_O_2_S (314.08), Calcd %: C, 57.32; H, 4.49; N, 17.80. Found %: C, 57.44; H, 4.52; N, 17.79.

*General procedures of compounds***9a***and***9b**. Refluxing equimolar amount of the *p*-chloropyrimidine derivative **3** with the appropriate 1^ry^ amine namely 5-*tert*-butylisoxazol-3-amine and 3-*tert*-butylisoxazol-5-amine, respectively, for 9 h in CHCl_3_ using 4 drops of TEA to afford compounds **9a** and **9b,** respectively.

*N-(5-tert-butylisoxazol-3-yl)-5,6,7,8-tetrahydrobenzo[4,5]thieno[2,3-d]pyrimidin-4-amine*: **9a**. Yield: 72%, m.p. 95–97 °C. ^1^H-NMR (400 MHz, DMSO-d6) ppm: δ 1.50 (m, 9H, t-butyl), 1.62–2.98 (m, 8H, cyclohexane), 2.13 (s, 1H, isoxazole ring), 4.00 (s, 1H, NH D_2_O exchangeable), 8.12 (s, 1H, C2-pyrimidine).; ^13^C-NMR (100MHz, DMSO-d6) ppm: δ 22.1, 22.4 24.0, 24.3, 32.2, 32.2, 33.3, 34.1, 97.4, 117.4, 126.3, 138.5, 145.4, 153.6, 157.1, 159.4, 161.3; Analysis for: C_19_H_20_N_4_OS (328): CHN calcd. C, 62.17; H, 6.14; N, 17.06; found: C, 62.14; H, 6.11; N, 17.02.

*N-(3-tert-butylisoxazol-5-yl)-5,6,7,8-tetrahydrobenzo[4,5]thieno[2,3-d]pyrimidin-4-amine*: **9b**. Yield: 75%, m.p. 110–112 °C. ^1^H-NMR (400 MHz, DMSO-d6) ppm: δ 1.50 (m, 9H, t-butyl), 1.62–2.98 (m, 8H, cyclohexane), 2.13 (s, 1H, isoxazole ring), 4.00 (s, 1H, NH D_2_O exchangeable), 8.12 (s, 1H, C2-pyrimidine).; ^13^C-NMR (100MHz, DMSO-d6) ppm: δ 23.5, 23.8, 24.3, 25.3, 32.3, 32.3, 32.3, 34.1, 97.4, 117.4, 126.3, 138.5, 145.4, 153.6, 157.1, 159.4, 161.3; Analysis for: C_19_H_20_N_4_OS (328): CHN calcd. C, 62.17; H, 6.14; N, 17.06; found: C, 62.14; H, 6.11; N, 17.02.

*General procedures of compounds***11a***and***11b**. Refluxing equimolar amount of the chloroacetamido thiophene derivative **10** with the appropriate 1^ry^ amine namely 5-*tert*-butylisoxazol-3-amine and 3-*tert*-butylisoxazol-5-amine, respectively, for 9 h in CHCl_3_ using 4 drops of TEA to afford compounds **11a** and **11b**, respectively.

*Ethyl 2-(2-(5-tert-butylisoxazol-3-ylamino)acetamido)-4,5,6,7-tetrahydrobenzo[b]thiophene-3-carboxylate*: **11a**. ^1^H-NMR (400 MHz, DMSO-d6) ppm: δ 1.62–2.98 (m, 8H, cyclohexane, 9H, t-butyl, 3H CH_3_-CH_2_), 2.13 (s, 1H, isoxazole ring), 3.80 (s, 1H, NH D_2_O exchangeable), 4.30 (m, CH_3_-CH_2_, CH_2_ acetamido), 8.12 (s, 1H, NH- D_2_O exchangeable); ^13^C-NMR (100MHz, DMSO-d6) ppm: δ 17.5, 20.1, 23.3, 24.2, 24.7, 25.3, 33.1, 33.1, 33.5, 57.5, 61.3, 95.9, 98.3, 128.4, 133.7, 154.4, 158.8, 161.1, 169.9, 178.0; Analysis for: C_20_H_27_N_3_O_4_S(405): CHN calcd. C, 59.24; H, 6.71; N, 10.36; found: C, 59.31; H, 6.69; N, 10.32.

*Ethyl 2-(2-(3-tert-butylisoxazol-5-ylamino)acetamido)-4,5,6,7-tetrahydrobenzo[b]thiophene-3-carboxylate*: **11b**. ^1^H-NMR (400 MHz, DMSO-d6) ppm: δ 1.62–2.98 (m, 8H, cyclohexane, 9H, t-butyl, 3H CH_3_-CH_2_), 2.13 (s, 1H, isoxazole ring), 3.80 (s, 1H, NH D_2_O exchangeable), 4.30 (m, CH_3_-CH_2_, CH_2_ acetamido), 8.12 (s, 1H, NH- D_2_O exchangeable); ^13^C-NMR (100MHz, DMSO-d6) ppm: δ 17.5, 20.1, 23.5, 23.8, 25.2, 32.3, 33.1, 33.1, 33.5, 57.5, 61.2, 96.6, 98.3, 128.4, 132.5, 153.6, 159.0, 161.3, 169.7, 177.2; Analysis for: C_20_H_27_N_3_O_4_S (405): CHN calcd. C, 59.24; H, 6.71; N, 10.36; found: C, 59.48; H, 6.78; N, 10.22.

### 3.3. Biology

#### 3.3.1. Antiproliferative/Cytotoxicity Assessment

##### Cell Culture

Three different cancer cells were used; colorectal cell line (HT-29), breast cancer cell line (MCF-7) and human hepatoma cell line (HepG-2) were obtained from Health Sciences Research Center, Riyadh, Saudi Arabia. All cell lines were cultured in their optimum media (DMEM or RPMI-1640 media) with 10% FBS, 100 U/mL penicillin, and 100 μg/mL streptomycin and passaged in a humidified incubator at 37 °C with 5% CO_2_. Cells were passaged at 80–90% confluence by trypsinization based on standard procedures.

##### Preliminary Antiproliferation Assay

*In*-*vitro* cytotoxicity screening was assessed by using MTT assay to select the promising synthesized compounds. The preliminary assay was performed at concentration 10 and 100 µM over HT-29, HepG-2 and MCF-7 cell lines. Cells were plated in 96-well plates (10^3^ cells/well) and allowed to attach for 24 h before treatment. Cells were exposed to compounds for 72 h, a vehicle control and control with no drug was included. After replacing the old media, 20 μL of 20 mM MTT (dissolved in PBS) was added and incubated for 3 h at 37 °C and 5% CO_2_. The MTT solution was carefully removed and 100 μL DMSO was added to solubilize the violet formazan crystals [39]. The data were expressed as the percentage of cells’ viability compared to untreated cells.

#### 3.3.2. Detailed Dose-Response Relationship against Cancer Cells

After assessment of the preliminary antiproliferative activity, dose-response curves of the compounds were used to calculate accurate IC_50_ values. Cells were exposed to the selected compounds for 72 h using doxorubicin as a reference standard.

#### 3.3.3. Kinase Screening

The potential inhibitory activity of the synthesized compounds on kinases was assessed using a homogeneous set of protein kinases extracted from HT-29 cell line. The screening assay was performed in a cell free system by using ADP-Glo assay (Promega, Madison, USA). Briefly, protein kinases were added into 96 well following by addition of the synthesized compounds at a single dose concentration 20 μM. After 45 min, the kinase reaction was stopped, and the remaining Adenosine Tri Phosphate (ATP) was depleted. Finally, the kinase detection reagent was added to convert ADP to ATP and allow the newly synthesized ATP to be detected using luminescent luciferase/luciferin reaction. The kinase inhibition data were expressed as the percentage of remaining kinase activity versus vehicle reaction.

##### FLT3 Inhibition

Inhibitory potency towards FLT3 enzyme was assessed to find compounds’ selectivity by using FLT3 Kinase Assay Kit (BD Biosciences, San Diego, CA, USA). The FLT3 (1.5 ng/µL) were incubated with serially diluted compounds for 45 min in a kinase reaction buffer. The FLT3 enzyme activity was measured by quantifying the amount of ADP produced during enzyme reaction. and the chemiluminescence was read by using Varioskan™ LUX multimode microplate reader (Thermo Scientific, Waltham, MA, USA). The IC_50_ was calculated with nonlinear regression using Prism version 8.4.3 (GraphPad).

#### 3.3.4. Apoptosis/Necrosis Assessment

Cell apoptosis was examined using an annexin V-FITC apoptosis detection kit (BD Pharmingen™, Franklin Lakes, NJ, USA) following the manufacturer’s protocol. briefly, HT-29 and HepG-2 cells were treated with the predetermined IC_50_s for 24 h and a drug-free medium-treated group was included as a control group. Post-treatment, cells were harvested, washed twice with ice-cold PBS, and resuspended in 500 μL of annexin V-FITC/PI solution for 30 min in the dark. After staining, cells were injected through CytoFLEX flow cytometry (Beckman Coulter, Brea, CA, USA). Ten thousand cells (gated events) were counted for each sample. Their mean fluorescence intensity was analyzed with Cytoexpert (Beckman Coulter).

#### 3.3.5. Autophagy Assessment

Acidic vesicular organelles (AVOs) were measured by acridine orange staining as reported [40]. After exposure to pre-calculated IC_50_ for 24 h, cells were trypsinized, washed twice with PBS, stained with 1 μg/mL Acridine Orange, and incubated in the dark for 30 min at room temperature. After incubation, cells were analyzed in the presence of the staining solution on Cyto FLEX flow cytometry (Beckman Coulter, Brea, CA, USA) using Cytoexpert software (Beckman Coulter).

## 4. Conclusions

The results of research are constantly being transformed into concrete advancements in diagnosis, prevention, and therapy, especially for cancer which represents one of the present ages’ greatest challenges. We were interested in synthesis and evaluating the biological cytotoxic activity of thieno[2,3-*d*]pyrimidine derivatives aiming to target the FLT3 kinase enzyme. A primary design strategy was built in reference to reported compounds with kinase inhibitory activity. Designed compounds were scanned for their affinity for kinases. The results were very promising with affinity ranges from 46.7% to 13.3%. Molecular docking studies were performed to investigate the affinity of the prepared compounds to the FLT3 kinase enzyme, which was expressed in an interesting value in the above-mentioned target-affinity prediction. The results of docking, regarding binding energy, and root mean square deviation RMSD in addition to the ligand interactions were promising. Compound **11** showed the lowest binding energy −9.01 kcal/mol while compound **5** expressed the lowest RMSD 0.79 **A** among the other compounds in relation to the co-crystalized ligand, amino acids involved in the main interactions between the protein ligand (P30) and the active site were also found to be in common between the synthesized compounds and the active ligand site. Compounds were then screened for their antiproliferative effects, and compounds **8** and **5** showed the most potent cytotoxic effects against all cell lines with IC_50_s ranging from 3.3 ± 0.90 μM to 15 ± 1.5 μM. Compound **8** resulted in significantly higher cytotoxic effect compared to the reference standard against MCF-7 with IC_50_s 4.132 ± 0.5 as well as HepG-2 with IC_50_s 3.3 ± 0.90. Further biological evaluation with performed to evaluate their induction of apoptosis and/or necrosis on HT-29 and HepG-2, Compounds **5**, **7a**, and **8** induced significant early apoptosis 65.3 ± 3.4%, 55.3 ± 0.70%, and 74.9 ± 4.9%, respectively) compared to untreated control HT-29 cells 9.6 ± 4.9%. Similarly, the exposure of HepG-2 cells to **5**, **7a**, **8**, and **9b** resulted in a significant increase in apoptosis 49.9 ± 3.5%, 25.8 ± 1.6%, 59.6 ± 0.19%, and 27.9 ± 2.9%, respectively, compared to untreated cells 15.4 ± 0.25%. Among all compounds examined, compound **5** induced a significant increase in the late apoptotic cell population in HepG-2 and HT-29 cells by 2.3 and 1.9-fold, respectively. None of the compounds induced any significant increase in necrotic cell population so we might conclude that the cytotoxic effect of these compounds against HepG-2 and HT-29 cells is mostly attributed to apoptosis rather than necrosis. Since the induction of apoptosis may trigger the autophagic cell death. We further investigated the effect of compounds **5**, **7a**, **8**, **9b**, and **10** on autophagy process within HT-29, HepG-2, and MCF-7 cells with flow cytometry. Analyzing the resulted in HT-29 cells, compounds **5**, **7a**, **9b**, and **10** significantly induced autophagic cells higher than untreated cells by 58.11 ± 2.8%, 53 ± 6.07%, 60.3 ± 0.4%, and 58.7 ± 2.4%, respectively. Compound **5** showed the highest autophagic induction among all compounds and significantly increased autophagic signal in HepG-2 and MCF-7 cells by 57.5 ± 16.8% and 44.07 ± 9.3%, respectively. The promising results prompted our interest for investigating the kinase activity, the potential inhibitory activity of the synthesized compounds on kinases was assessed. Initial screening for all compounds showed inhibition activity ranging from 41.4% to 83.5%. Compounds that recorded significant activity with ≥77% inhibition were then further investigated for their specific FLT3 kinase inhibition using FLT3 kinase kits. Compounds **5**, **8**, **9b**, and **10** recorded kinase inhibition 80.29%, 79.35%, 81.8%, and 79.19%, respectively. Interestingly, compound **5** exhibited the highest inhibitory activity against FLT3 with IC_50_ 32.435 ± 5.5 μM.

According to the aforementioned findings, we conclude that compounds **5**, **7a**, **8**, and **9b** are promising cytotoxic newly synthesized thienopyrimidines targeting kinases, especially among which compound **5** that also showed the potential to activate autophagic and apoptotic cell death in cancer cells. Moreover, based on the bioinformatics data analysis, all of the investigated compounds are considered good candidates for “drug-like” molecules with promising bioavailability.

## Figures and Tables

**Table 1:** Compounds’ binding energy and root mean square deviation RMSD in addition to the ligand interactions.

Compound	Binding Energy Kcal/mol	RMSD (A)	2D-Interactions	Amino Acids Involved in Interaction
**5**	−8.068	0.792	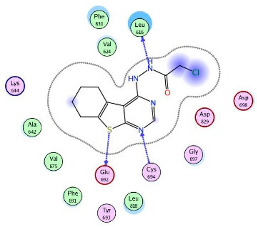	Leu 616 Cys 694 Glu 692
**8**	−7.529	1.044	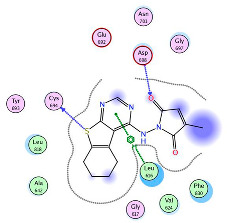	Leu 616 Cys 694 Asp 698
**9b**	−8.360	1.051	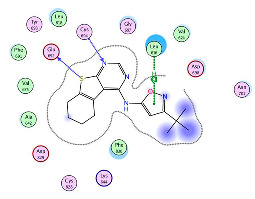	Leu 616 Cys 694 Glu 692
**10**	−8.0461	1.828	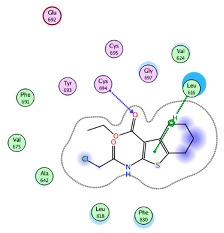	Leu 616 Cys 694
**11b**	−9.072	1.512	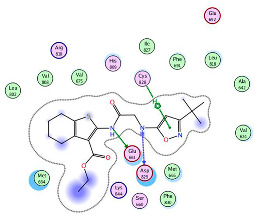	Glu 661 Asp 829 Cys 828
**P30**	−10.651	1.247	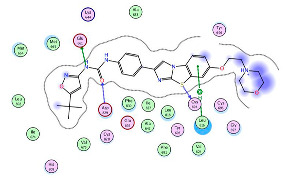	Glu 661 Asp 829 Leu 616 Cys 694

**Table 2:** IC_50_ in µM of the tested compounds on HT-29, MCF-7, and HepG-2 cell lines.

Compound No.	Anti-Proliferative Activities in Different Cancer Cell Lines IC50s (μM)		
	HT-29	HepG-2	MCF-7
**5**	10.1 ± 0.4	5.3 ± 1.6	7.3 ± 4.5
**7a**	15.7 ± 0.4	8.0 ± 0.04	15.0 ± 0.8
**8**	15 ± 1.5	3.3 ± 0.9	4.1 ± 0.5
**9b**	28.0 ± 13.1	6.9 ± 2.7	35.3 ± 1.5
**10**	17.1 ± 0.7	7.5 ± 0.09	16.4 ± 5.8
**Dox**	1.4 ± 1.16	13.915 ± 2.2	8.43 ± 0.5

**Table 3:** IC_50_ values in µM reflecting FLT3-kinase activity after exposure to selected compounds.

Compound No.	IC_50_ (µM)
**5**	32.43 ± 5.5
**8**	40.55 ± 6.3
**9b**	39.61 ± 4.6
**10**	40.04 ± 5.5

Data are expressed as mean ± SD; *n* = 3.

**Table 4:** Bioinformatics assessment for compounds with best biological and/or modeling results.

Compound #	Number of HBA	Number of HBD	Log *P*	TPSA	BBB Score	Drug Likeness Score	Compounds in Relation to Drug and Non-Drug Scores
**5**	4	2	2.32	58.87 A2	4.18	0.63	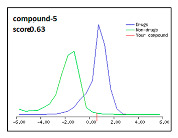
**8**	5	1	2.37	63.74 A2	3.84	0.98	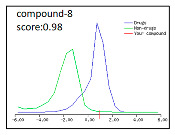
**9**	5	1	5.07 (>5)	53.18 A2	4.54	0.73	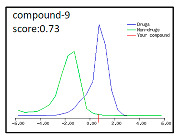
**10**	4	1	2.96	43.86 A2	4.23	0.48	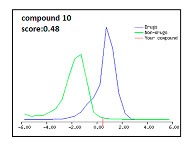
**11**	6	2	4.55	76.64 A2	3.20	0.69	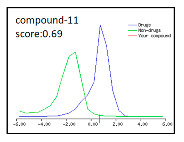

## Future Investigations

Prepared compounds are promising cytotoxic candidates that needs to be biologically tested on normal cells to determine their selectivity and further in-vivo future investigations if applicable.
